# A broad neutralizing nanobody against SARS-CoV-2 engineered from an approved drug

**DOI:** 10.1038/s41419-024-06802-7

**Published:** 2024-06-28

**Authors:** Qianyun Liu, Yuchi Lu, Chenguang Cai, Yanyan Huang, Li Zhou, Yanbin Guan, Shiying Fu, Youyou Lin, Huan Yan, Zhen Zhang, Xiang Li, Xiuna Yang, Haitao Yang, Hangtian Guo, Ke Lan, Yu Chen, Shin-Chen Hou, Yi Xiong

**Affiliations:** 1 State Key Laboratory of Virology, Department of Thoracic Surgery, Renmin Hospital of Wuhan University, Wuhan, 430060 China; 2 Shanghai Institute for Advanced Immunochemical Studies and School of Life Science and Technology, ShanghaiTech University, Shanghai, 201210 China; 3 Lingang Laboratory, Shanghai, 200031 China; 4 Shanghai Clinical Research and Trial Center, Shanghai, 201210 China; 5 Bioduro-sundia LLC., Wuxi, 214174 Jiangsu China; 6 State Key Laboratory of Virology, Modern Virology Research Center and RNA Institute, College of Life Sciences and Frontier Science Center for Immunology and Metabolism, Wuhan University, Wuhan, 430072 China; 7 Animal Biosafety Level-III Laboratory/Institute for Vaccine Research, Wuhan University, Wuhan, 430071 China; 8 The State Key Laboratory of Pharmaceutical Biotechnology, School of Life Sciences, Nanjing University, Nanjing, 210023 Jiangsu China; 9 Bayray Innovation Center, Shenzhen Bay Laboratory, Shenzhen, 518107 Guangdong China

**Keywords:** Electron microscopy, Viral infection

## Abstract

SARS-CoV-2 infection is initiated by Spike glycoprotein binding to the human angiotensin-converting enzyme 2 (ACE2) receptor via its receptor binding domain. Blocking this interaction has been proven to be an effective approach to inhibit virus infection. Here we report the discovery of a neutralizing nanobody named VHH60, which was directly produced from an engineering nanobody library based on a commercialized nanobody within a very short period. VHH60 competes with human ACE2 to bind the receptor binding domain of the Spike protein at S^351^, S^470-471^and S^493-494^ as determined by structural analysis, with an affinity of 2.56 nM. It inhibits infections of both ancestral SARS-CoV-2 strain and pseudotyped viruses harboring SARS-CoV-2 wildtype, key mutations or variants at the nanomolar level. Furthermore, VHH60 suppressed SARS-CoV-2 infection and propagation 50-fold better and protected mice from death for twice as long as the control group after SARS-CoV-2 nasal infections in vivo. Therefore, VHH60 is not only a powerful nanobody with a promising profile for disease control but also provides evidence for a highly effective and rapid approach to generating therapeutic nanobodies.

## Introduction

The ongoing COVID-19 pandemic, caused by the novel Severe Acute Respiratory Syndrome Coronavirus 2 (SARS-CoV-2), has persisted for years, resulting in significant loss of life and profound impacts on global economic and social activities. With over 700 million confirmed infections and 6 million reported deaths worldwide, along with enduring physical and mental health consequences, the toll of this pandemic has been staggering [1–6]. With the deployment of efficacious vaccines and drugs, virus transmission and modality have dropped drastically, and the battle against COVID-19 has shifted from epidemic prevention to the development of underlying basic sciences and advanced technologies arising from this tragic event, to gear up for the next unpredictable pandemic [7]. However, accumulating evidence indicates that the virus can infect multiple organs and persist or reinfect due to rapid mutations inherent in RNA viruses. This genetic variability allows the virus to evade existing drug targets and manipulate the host immune system [8–12]. New variants continue to emerge, posing relentless challenges to public health. Therefore, there is an urgent need for further efforts to comprehensively understand the pathogenic mechanisms of viral infection and evasion and to develop timely new prevention and treatment strategies.

SARS-CoV-2 is a single-stranded RNA virus with a genome of approximately 30,000 nucleotides, encoding 29 proteins from 14 functional open reading frames, including 4 structural proteins, 16 nonstructural proteins, and 9 accessory proteins [13, 14]. The viral infection is mediated by structural proteins, notably the glycoprotein Spike (S), which binds to the host receptor Angiotensin Converting Enzyme 2 (ACE2). The S protein, a trimeric fusion protein on the virion surface, undergoes cleavage into receptor-binding fragment S1 and fusion fragment S2 upon engagement with the host cell, facilitated by cellular serine protease TMPRSS2 and lysosomal proteases cathepsins [15, 16]. Additionally, the proprotein convertase Furin has been implicated in SARS-CoV-2 entry by pre-activating the S protein [16]. S1 firstly interacts with ACE2 through the receptor binding domain (RBD) on its C-terminus, then switches its conformation from “sitting-down” to “standing-up” to dissociate and expose S2 which facilitates virus fusion with the cell membrane. Compared to SARS-CoV, SARS-CoV-2 S protein demonstrates a stronger affinity for ACE2 due to amino acid differences at the S1/S2 cleavage site, contributing to its heightened transmissibility [16, 17]. Extensive analyses at the atomic level have elucidated the structure of the S protein, its binding pattern, and its dynamic conformational changes during virus entry and fusion, providing valuable insights for antiviral drug development [17–20]. However, the evolution of SARS-CoV-2 has given rise to numerous variants, each with distinct characteristics that pose challenges to control and prevention efforts [10, 11, 21]. Spike protein mutations contributed greatly to such a predicament. Mutations in the Spike protein, such as E484K [22, 23], N501Y [24], D614G [25, 26], or combinations of mutations in variants like B1.1.7 (U.K. variant), B.1.351 (South African variant), P.1. (Brazilian variant), B.1.525 (Nigeria variant), B.1.617.2 (Delta variant), Omicron and Omicron-related lineages (BA.1 to BA.5, XBB, EG.5, etc), have been associated with enhanced binding to ACE2, increased viral replication and transmission, and evasion of neutralizing antibodies and vaccines. This ongoing evolution underscores the urgency of developing new therapeutics and vaccines to combat COVID-19 [22, 24, 25, 27–33].

Among various monoclonal antibodies derived from human or small laboratory animals, nanobodies represent a distinct class with unique properties [34–36]. Nanobodies, also known as VHH, are single-domain antibody fragments originally found in llamas and other camelids. Unlike conventional IgG antibodies, which consist of covalently linked two light chains and two heavy chains, nanobodies contain only the monomeric target recognition module of the heavy chain, while retaining similar specificity and affinity as IgG antibodies. With a small size of only 15 kD, nanobodies are easier to produce and manipulate, offering robust thermostability, solubility, and permeability, yet much low immunogenicity [37]. Nanobodies have been extensively developed as therapeutic agents for cancer, autoimmune disorders, and renal diseases, with one nanobody approved by the FDA for clinical use, underscoring the potential of single-domain constructs as a novel drug platform [38].

Traditionally, antibody generation and screening involve animal immunization or the use of contagious samples from patients, posing risks to researchers exposed to infectious pathogens and requiring significant time for the immunization process. To address these challenges during a global emergency, we describe the rapid engineering of a potent nanobody with broad neutralizing activity against SARS-CoV-2. Leveraging the commercialized nanobody caplacizumab, targeting the von Willebrand factor and approved by the EMA in 2018 and subsequently by the FDA in 2019, we swiftly constructed and screened an engineering phage library against the RBD. From this effort, we identified a nanobody named VHH60, derived from caplacizumab (a VHH targeting von Willebrand factor, approved by EMA in 2018 and later by the FDA in 2019) specific to the RBD, which binds to the RBD with single-digit nanomolar affinity. VHH60 effectively blocks the entry of both wild-type viruses and variants, protecting host cells and mice from viral infection. These findings highlight VHH60 as a promising candidate for further investigation in combating COVID-19. Moreover, our results illuminate the potential for safely and promptly discovering therapeutic nanobodies in response to emerging viral threats.

## Results

### Generation of neutralizing nanobodies against SARS-CoV-2

**Fig. 1:**
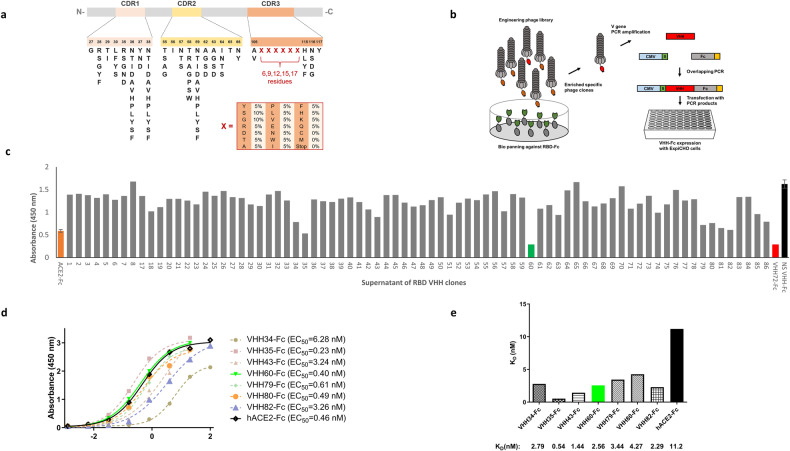
Screen and identify RBD-specific nanobodies. **a** Schematic of nanobody engineering. Sequence of the framework for caplacizumab are fixed (gray); the CDR1, CDR and CDR3, shown in pink, yellow and orange, respectively, are engineered for RBD specificity. The amino acid diversity of each position in the CDRs are indicated by one letter codes, that of highly variable loop in CDR3 is indicated by “X” representing the amino acid composition in the box. The residue numbers indicate the length diversity of highly variable loop in CDR3. **b** The scheme of screening. The caplacizumab engineering phage library was subjected to 3 rounds of solid phase biopanning against RBD-Fc, the RBD-specific VHHs were recombinantly expressed by PCR-based gene expression cassette strategy in high-throughput format. **c** Blocking ELISA to select nanobodies that can block hACE2 binding to RBD, smaller value reflects stronger blockage. Orange bar: hACE2-Fc as an internal control; Red bar: VHH72 as a positive control; Black bar: Non-reactive nanobody as a negative control. **d** EC_50_ from ELISA-based binding assay in (**c**). **e** chart of SPR results from Fig. S2a.

To generate nanobodies capable of neutralizing SARS-CoV-2, we employed the framework of caplacizumab as a template and engineered the complementary determining regions (CDRs) to construct a library for screening caplacizumab mutants specific to SARS-CoV-2. The amino acid diversity of CDR1, CDR2, and the flanking region of CDR3 was designed based on the natural VHH repertoire. At each position of the highly diversified region in CDR3, 18 amino acids were included, with frequencies of 5% for Y, R, D, T, A, P, L, V, E, N, W, I, F, H, K, Q; 10% for S and G; while stop codons, C, and M were excluded (indicated as X in Fig. 1a). In addition, length diversity was introduced in the highly diversified CDR3 loop, ranging from 6 to 17 residues. The designed amino acid diversity was genetically engineered into the CDRs of caplacizumab using a high-speed DNA mutagenesis method (Fig. 1a).

The resulting nanobody mutants were displayed on the phage surface as a library at a size of 10^10^ followed by 3 rounds of selections against the recombinant RBD domain of the S protein. RBD-specific clones were identified through single-clone phage ELISA with positive signals. After sequencing, 78 unique VHH genes were identified and subsequently amplified by PCR from the phage. Next, the mammalian expression cassette was assembled with a CMV promoter and an Fc domain of human IgG1 fragment on the VHH’s 5’ and 3’ end, respectively, using a second-round overlapping PCR. The final PCR products were transfected into ExpiCHO cells for Fc-tagged nanobody expression in a high-throughput manner using multiwell plates (Fig. 1b). Next, the cell culture medium containing Fc-tagged nanobodies was utilized to compete with Fc-tagged human ACE2 (hACE2) for binding to the RBD as the second-round ELISA-based blocking screening (Fig. 1c). Finally, 7 nanobodies were identified and displayed blocking ability similar to that of the positive control VHH72-Fc [39]. All 7 antibodies were expressed for further characterization. After protein A column purification, Fc-tagged nanobodies were present as monomers of ~40 kD in reducing gels, with an estimated purity of 90% (Fig. S1).

### Mutant nanobodies specifically bind to SARS-CoV-2 RBD with high affinity

We conducted ELISA to evaluate the binding capacity of the nanobodies to their original target RBD protein in a concentration-dependent manner. VHH35, VHH60, VHH79, and VHH80 showed slightly higher or comparable affinity against the recombinant RBD protein compared to hACE2-Fc (Fig. 1d). Of all the identified nanobodies, VHH35 displayed the highest affinity with an EC_50_ of 0.23 nM. We then analyzed the affinity by surface plasmon resonance (SPR). VHH35 consistently exhibited the lowest K_D_ of 0.54 nM for RBD, while the rest of the nanobodies were all bound to RBD with single-digit nanomolar K_D_ values (Figs. 1e and S2a). To confirm the blocking effect of nanobodies on the interaction between RBD and hACE2, we used SPR to measure the binding of hACE2 to RBD after the RBD was preoccupied with nanobodies captured by protein A on the chip. Our data indicated that after RBD was bound by all the tested nanobodies (including VHH34, VHH35, VHH43, VHH60, VHH79, VHH80, VHH82), no obvious binding curve of hACE2 to RBD could be detected, further demonstrating that these nanobodies have blocking effect on the binding of RBD to hACE2 (Fig. S2). Together, the ELISA and SPR data indicated that these 7 nanobodies from the blocking screening have the ability to compete with the binding of hACE2 to RBD.

### VHH60 suppresses SARS-CoV-2 virus infection and amplification in vitro and in vivo

**Fig. 2:**
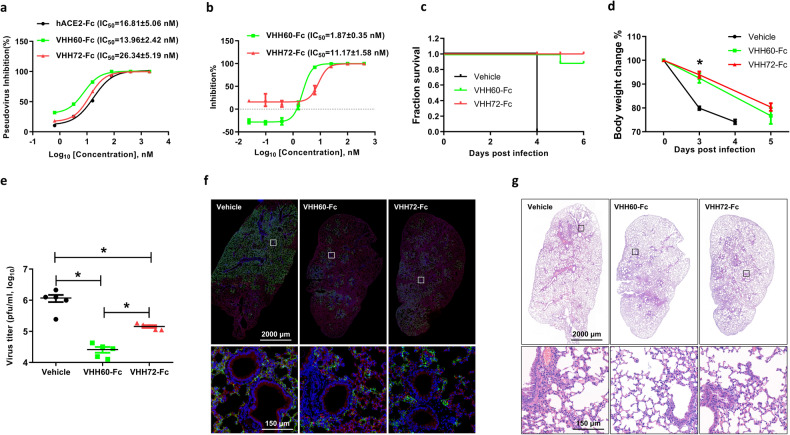
Antiviral activity of VHH60. **a** VHH60 inhibits pseudovirus carrying wildtype spike protein infection on Caco-2 cell line. **b** VHH60 inhibits ancestral SARS-CoV-2 infection on Vero-E6. **c** Survival curve of mice infected by SARS-CoV-2. Vehicle group all died at 4 d.p.i (5/5), one in VHH60 group (1/5) died. **d** Body weight change of mice infected by SARS-CoV-2. Data is represented as ratio of body weights at indicated timepoint versus day 0 (*n* = 10 at 0 and 3 d.p.i, *n* = 4 at 4 d.p.i, *n* = 5 at 5.d.p.i). **e** Virus load in lung after 3 days of infection (*n* = 5). **f** Representative image of immunofluorescence from lung. Blue: Nuclei, Red: ACE2, Green: nucleocapsid protein (NP). **g** Representative image of hematoxylin-eosin (H&E) staining from the lung. Scar bar, 2000 μm in the up row, 150 μm in the down row. One-way ANOVA with Tukey’s multiple comparison test was used. *P < 0.05. Error bars represent the means with SDs.

We investigated the neutralizing activity of VHH35 and VHH60 using a pseudovirus-based cell entry assay. Pseudovirus bearing the S protein and luciferase was incubated with various concentrations of nanobodies for 30 min before infecting Caco-2 cells. The resulting luciferase activity measured at 48 h post-infection suggested that VHH60 offered the best protection compared to VHH72 and ACE2, with IC_50_ values of 13.96 ± 2.42 nM, 26.34 ± 5.19 nM, and 16.81 ± 5.06 nM, respectively (Fig. 2a). Surprisingly, VHH35 did not show expected efficacy, with an IC_50_ value of around 100 nM (Fig. S3). This discrepancy suggests that binding affinity was not absolutely correlated with antiviral activity, as observed by others [40].

To further evaluate the antiviral effect of VHH60, we used ancestral SARS-CoV-2 virus to test it on Vero-E6 cells in vitro. Virus was premixed with serially diluted nanobodies for 30 min and then added to the Vero-E6 cells to propagate for 24 h. Viral RNA level was measured by RT-PCR. The data showed that VHH60 inhibited viral infection and propagation with an IC_50_ of 1.87 ± 0.35 nM, which was 6-fold lower than the IC_50_ of the reference VHH72 (11.17 ± 1. 58 nM) (Fig. 2b). Encouraged by this result, we then investigated the antiviral potential of VHH60 in vivo (Fig. S4). The female K18-hACE2 transgenic mice expressing human ACE2 were randomly grouped (10 mice per group) and intraperitoneally administered nanobodies or controls (Vehicle: PBS) at 0.5 mg/kg 24 h before inoculation with ancestral SARS-CoV-2 virus intranasally. Five mice of each group were sacrificed for pathological analysis at 3 days post-infection (d.p.i.) as planned (Fig. S4). All the remaining mice in the vehicle group died at 4 d.p.i. (5 out of 5, observed on day 5), but mice treated with the nanobodies (VHH60 and VHH72) survived up to 6 days, except for one mouse in the VHH60 group that died at 5 d.p.i. (1 out of 5, observed on day 6) (Fig. 2c). All VHH60- and VHH72-treated mice were sacrificed at day 6 post-infection due to the body weight of the mice had dropped by up to 20%, according to the termination criteria of IACUC protocol. Consistent with previous reports that virus infection could cause body weight loss, we also observed that at 3 d.p.i., the body weight of mice from the vehicle group had dropped by 20% at 3 d.p.i. In contrast, the body weight of mice treated with VHH60 and VHH72 decreased only slightly (Fig. 2d). To assess the protective effect more accurately, viral load was evaluated at 3 d.p.i. when all mice were still alive. Virus titer from the lungs in the VHH60-treated group was significantly suppressed to a level 45-fold lower than that of the vehicle and 9-fold lower than that of the VHH72 group, respectively (Fig. 2e). Immunofluorescent data clearly confirmed that the viral particles, represented by green nucleocapsid staining, were much fewer in the VHH60 and VHH72 groups compared to those in the vehicle group (Fig. 2f). We did not observe any significant difference in the red signal from ACE2 staining, which could exclude the possibility that virus titer was affected by the ACE2 level. Further histological analysis of the lungs was performed. The infection of SARS-CoV-2 caused severe interstitial pneumonia characterized by a large number of inflammatory cells infiltrating the lungs and the thickening and rupture of the alveolar septum in the vehicle group mice. Meanwhile, relatively minor lung damages were observed in mice treated with VHH60 or VHH72 (Fig. 2g). Together, the results strongly support that VHH60 is highly effective in restraining SARS-CoV-2 infection and proliferation both in vitro and in vivo, ameliorating disease progression and improving health.

### VHH60 blocks the infection of mutated pseudovirus

**Fig. 3:**
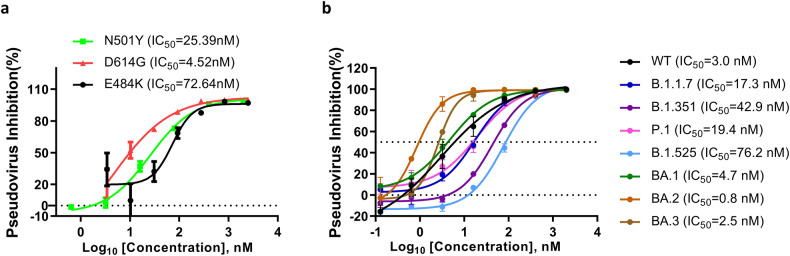
VHH60 blocks escape mutants and prevailing variants. **a** VHH60 inhibits pseudovirus carrying spike protein with a single mutation from infecting Caco-2 cell line. **b** VHH60 inhibits pseudovirus carrying spike protein with multiple mutations as in reported variants from infecting Caco-2 cell line. Error bars represent the means with SDs.

Given the high mutagenic capacity of SARS-CoV-2 as an RNA virus, mutations and variants resistant to current antibodies or vaccines have been studied and described [22, 24–30]. Antibody cocktails or broadly neutralizing antibodies have stood out and gained more attention as new modalities to combat COVID-19 [41, 42]. In addition to the wildtype S protein, mutants and variants carrying more than one mutation were tested with VHH60 in pseudoviral entry assays. VHH60 inhibited all single mutants E484K, N501Y, and D614 at the nanomolar IC_50_ level (Fig. 3a). Strikingly, VHH60 also exhibited robust activity to suppress several Omicron sub-variants (BA.1, BA.2, and BA.3) at nanomolar levels, close to or even better than that for the wildtype S protein (Fig. 3b).

### The epitopes for VHH60

**Fig. 4:**
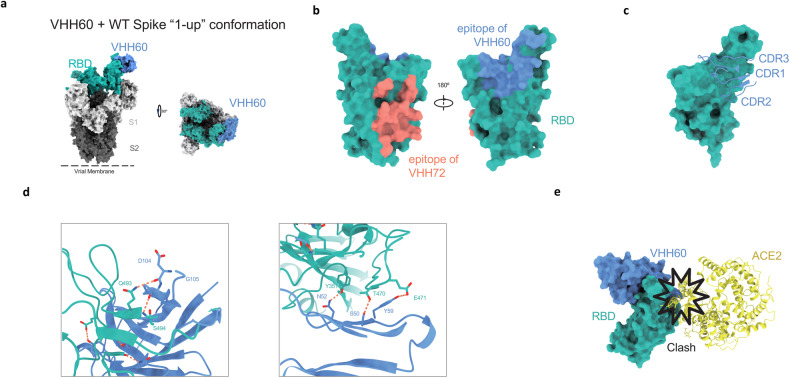
Structures of VHH60 binding to SARS-CoV-2 spike protein. **a** Cryo-EM for the Spike-VHH60 complex observed to a resolution of 3.79 Å, revealing binding of VHH60 to RBDs in the “1-up” conformation. **b** Comparison of the epitope between VHH60 (light sea green) and VHH72 (salmon, PDB ID: 6WAQ). **c** CDR loops of VHH60 (cornflower) overlaid on the surface epitope (salmon) representation of RBD (light sea green). **d** The hydrogen bonds at the binding interface between VHH60 and SARS-CoV-2 RBD based on the crystal structure of the complex at 3.40 Å. **e** Superposition of the RBD-ACE2 model (PDB ID:7XO9) to that of the VHH60- RBD model shows there is a steric hindrance between VHH60 and ACE2.

To further investigate the mechanism of VHH60 inhibitory effect on not only SARS-CoV-2 WT but also other variants, the single-particle cryo-electron microscopy (cryo-EM) structures was determined to illustrate the nanobody VHH60 in complex with the prefusion-stabilized SARS-CoV-2 WT variant S trimer [43] (Fig. S7). When VHH60 are bound to the WT Spike, the trimers adopted the “1-up” conformation (Figs. 4a, S7). We refined the conformation to an overall resolution of 3.79 Å. This image represents a single VHH60 bound to a single up RBD, while the down RBDs remains in an apo form.

To further investigate the detailed interaction between VHH60 and RBD, the crystal structure of the complex was solved to 3.40 Å. Two complementarity determining regions (CDRs; CDR2, CDR3) of VHH60 are involved in interaction with the S^351^, S^470-471^and S^493-494^ (Fig. 4b, c). Some potential hydrogen bonds were found on the interface of VHH60 and RBD, representing the unique interaction network between the VHH60 CDRs and the residues within the epitope in the WT RBD (Fig. 4c, d). The epitopes on the RBD include Y351, T470, E471, Q493, and S494, most of which are conserved among the epidemic variants (except Q493R, a mutation was first founded in VOC Omicron (B.1.1.529), but reversion mutation R493Q was founded in the sublineages Omicron BA.4/ BA.5) (Fig. 4d) [44].

Most of the epitopes of VHH60 overlapped with ACE2 binding site S^437-508^, and the superimposition of the RBDs in the structures of VHH60-RBD complexes indicates a minor steric clash between ACE2 and VHH60 bound with RBD, which explains the partial competition between VHH60 and ACE2 for WT S trimer binding (Fig. 4e).

## Discussion

Overall, our results demonstrate a method for generating neutralizing antibodies against the pandemic SARS-CoV-2 within a remarkably short timeframe. This includes template synthesis, vector construction, library construction, panning, single clone screening, VHH-Fc expression, and validation. Unlike conventional antibody discovery methods involving immunized animals, this strategy saves 2 to 3 months of immunization time, and using commercialized antibodies as templates can reduce the developability risks by their proven clinical framework. Our proposed method is especially suitable for developing therapeutic agents against future pandemic infectious diseases.

Nanobodies represent a significant innovation in antibody-based therapies. Unlike traditional monoclonal antibodies, nanobodies offer natural advantages for prophylactic applications, particularly relevant considering SARS-CoV-2’s predominant mode of transmission via droplets and aerosols [45].

Multivalent nanobodies are known to potentially exhibit even higher affinity and antiviral activity. Thus, we generated a His-tagged trimeric VHH60 and assessed its antiviral activity. Compared to its monomeric form, the Tri-VHH60-His construct demonstrated a 9-fold increase in potency in blocking ACE2-RBD interaction, with an IC_50_ of 33.57 nM (Fig. S5). Consequently, Tri-VHH60-His also exhibited superior inhibition of pseudovirus infection, outperforming monomeric VHH60 by 9-fold (Fig. S6). Clearly, VHH60 can serve as a fertile foundation for further optimization, especially for elderly people and immunocompromised patients.

The potent neutralizing activity of VHH72 highlights the significant clinical potential of nanobodies derived from llamas and other camelids [39]. Notably, VHH72 recognizes epitopes on the inner side of the RBD, distinct from the epitope targeted by VHH60 (Fig. S8). This advantage, coupled with the small size of nanobodies, facilitates the synergistic use of multiple nanobodies with different specificities, potentially leading to enhanced efficacy. Throughout the COVID-19 pandemic, over 300 nanobodies from various sources have been evaluated for their antiviral activities. Some have demonstrated affinities as high as femtomolar to RBD in in vitro assays [46], suggesting the potential for nanobodies to evolve into versatile medications against COVID-19. In this context, the VHH60 nanobody presented here shows excellent potential for further optimization and development for clinical applications, given its broad neutralizing capacity against both wildtype and most reported mutant forms of the SARS-CoV-2 virus. Additionally, we have demonstrated the advantages of an engineered antibody library over traditional ones, enabling the rapid discovery of functional antibodies without the lengthy processes of animal immunization and exposure to hazardous pathogens. The nanobody engineering platform described here can be readily deployed for discovering antiviral molecules in response to future viral emergencies.

## Material and methods

### Cell lines and viruses

Vero-E6 (ATCC® CRL-1586), Caco-2 (ATCC® HTB-37), and 293 T (ATCC® CRL-3216) cells were cultured in Dulbecco’s Modified Eagle Medium (DMEM) supplemented with 10% fetal bovine serum (FBS) (Cat. # 26140079, Thermo Fisher) and 1% antibiotic-antimycotic (Cat. # 15240096, Thermo Fisher) at 37 °C with 5% CO_2_. The ExpiCHO Expression System was obtained from Thermo Fisher (#A29133), and the SARS-CoV-2 Virus was provided by Dr. Yu Chen from Wuhan University.

### Protein expression and purification

The constructs of VHHs were selected from phage display, RBD fragment (aa319-541) of SARS-CoV-2 S protein (GenBank: MN908947.3) was synthesized by Genewiz Inc, the extracellular domain of human ACE2 (1-740 aa) (GenBank: NM_021804.1) was amplified from a plasmid (Cat. # HG10108-ACG, Sinobiologic). The whole coding cassette was ligated into a pCMV3 expression vector with a signal peptide of MEFGLSWVFLVALFRGVQC at the N-terminal, and either a 6-his tag (for RBD-his) or a human IgG1 Fc fragment with (GSSSS)^3^ linker at C-terminal. For Fc-tagged protein, the construct was expressed in 293 F cells by transfecting with PEIMAX PEI MAX™ (Cat. # 24765-1, Polysciences) according to the manual. The protein was purified from culture supernatant by protein-A affinity chromatography and was buffer changed into PBS and stored at −70 °C. For His-tagged protein, the construct was used for expression in CHO cells using ExpiCHO^TM^ Expression according to the manual. The protein was purified from culture supernatant by Nickel affinity chromatography and finally dissolved in PBS (pH 7.4) and stored at −70 °C. For VHH-Fc, the protein was purified from culture supernatant by protein A affinity chromatography and was buffer changed into PBS and stored at −70 °C. Extra polishing by hydroxyapatite chromatography was added to achieve HPLC purity of over 95% for specific experiments.

For protein A affinity chromatography, the culture supernatant from the transient expression product was clarified by centrifuge at 3000 g for 10 min and was mixed with an equal volume of 1.5 M Glycine, 3 M NaCl, pH 8.9. the protein was purified with HiTrap^TM^ MabSelect^TM^ SuRe^TM^ column (Cat. # 11003494, Cytiva), and was eluted with 20 mM acetic acid, pH3.5. The acid eluted fraction was neutralized with 1 M Tris-HCl, pH9.0 and was concentrated and desalted into PBS with Amicon^®^ Ultra-15, PLTK Ultracel-PL membrane (Millipore Sigma) with appropriate MWCO.

For Nickel affinity chromatography, the culture supernatant from transient expression product was clarified by centrifuge at 3000 g for 10 min and was mixed with equal volume of 20 mM imidazole, 500 mM NaCl, 20 mM Tris pH8.0. the protein was purified with HisTrap^TM^ HP column (Cat. # 17524701, Cytiva), and was eluted with 500 mM imidazole, 500 mM NaCl, 20 mM Tris pH8.0. The eluted fraction was concentrated and desalted into PBS with Amicon® Ultra-15 centrifugal unit (Millipore Sigma) with appropriate MWCO.

For polishing with hydroxyapatite chromatography, the sample was buffer changed into 5 mM sodium phosphate, 20 mM MES, pH6.6, and was loaded on self-packed columns with Ca^++^Pure HA resin (Cat. # 45039, Tosoh Bioscience); the protein was eluted by gradient elution with 400 mM sodium phosphate, 20 mM MES, pH6.6, and the targeted fraction was concentrated and desalted into PBS with Amicon® Ultra-15 centrifugal unit with appropriate MWCO. All proteins were checked by SDS-PAGE and HPLC with TSKgel G3000SWXL (Cat. # 08541, Tosoh Bioscience) for purity.

To purify SARS-CoV-2 RBD (319–531), the encoding construct was synthesized by Genwiz and inserted into a modified pFastBac1 vector, followed by a 3 C cleavage site and 6-his-tag at the C-terminal and a GP64 signal peptide at N-terminal. The purification procedure was followed by Yang et, al. [47]. In brief, Sf9 insect cells were infected with a high-titer recombinant baculovirus expressing RBD and incubated for 72 h at 27 °C for protein expression. The culture supernatant was collected after centrifugation and incubated with Ni Sepharose excel (Cat. # 17371203, Cytiva) at 4 °C for 2 h to capture His-tagged RBD. After washing with 20 mM imidazole, samples were eluted with a buffer containing a 300 mM imidazole concentration yielded the target protein. Enzymatic digestion with custom-made Precision Protease and PNGaseF removed the His-tag and N-linked glycans, respectively. An anion ion exchange chromatography was applied to remove the enzymes. Finally, the purified RBD was collected and analyzed by SDS-PAGE.

### Construction of phage displayed engineering VHH library by oligonucleotide-directed mutagenesis

To create a diverse VHH library, we employed oligonucleotide-directed mutagenesis to target three complementarity-determining regions (CDRs) of caplacizumab. Each CDR was subjected to mutagenesis using synthesized oligonucleotides designed to introduce tailored diversity in amino acids. Mixed nucleotides with degenerate codons were utilized for CDR1 and CDR2, while CDR3, with its highly variable loop, was synthesized using a trimer phosphoramidite mixture. The mutagenic oligonucleotides for each CDR were first mixed and phosphorylated using T4 polynucleotide kinase (New England BioLabs) in a reaction mixture containing 70 mM Tris–HCl (pH 7.6), 10 mM MgCl2, 1 mM ATP, and 5 mM dithiothreitol (DTT) at 37 °C for 1 h. Next, a single-stranded m13mp2 DNA template containing uracil was obtained from defective E. coli. The phosphorylated oligonucleotides were annealed to the single-stranded DNA template at a molar ratio of 3:1 (oligonucleotide: ssDNA) by heating the mixture at 90 °C for 2 min, followed by a progressive temperature decrease of 1 °C/min to 20 °C in a thermal cycler. Subsequently, the template-primer annealing mixture was incubated in a solution containing 0.32 mM ATP, 0.8 mM dNTPs, 5 mM DTT, 600 units of T4 DNA ligase, and 75 units of T7 DNA polymerase (New England BioLabs) to prime in vitro DNA synthesis. After overnight incubation at 20 °C, the synthesized double-stranded DNA (dsDNA) was desalted and electroporated into E. coli ER2738 at 3000 V, followed by infection with the M13KO7 helper phage and overnight culturing. Finally, the phage displaying nanobodies as a library in the culture medium was harvested and precipitated using polyethylene glycol (PEG)/NaCl for further use. Typically, 1 μg of dU-ssDNA produced about 10^7^–10^8^ recombinant phage variants, with 75–90% of the phage variants carrying mutagenic oligonucleotides in all three CDR regions simultaneously.

### Screening of anti-RBD nanobodies

RBD-specific VHHs were identified from the screening (bio panning) of the phage-displayed engineering VHH library. Recombinant RBD-Fc (2~5 μg per well) was coated in PBS buffer (pH7.4) in NUNC 96-well Maxisorb immunoplates overnight at 4 °C and then blocked with 2% BSA in PBST for 1 h. After blocking, 100 μL of resuspended polyethylene glycol (PEG)/NaCl-precipitated phage library (10^13^ cfu/mL in blocking buffer) was added to each well for 1 h under gentle shaking. The plate was washed 10 times with 200 μL PBST [0.05% (v/v) Tween20] and 2 times with 200 μL PBS. The bound phages were eluted with 100 μL of 0.1 M HCl/glycine (pH 2.2) per well and immediately neutralized with 8 μL of 2 M Tris-base buffer (pH9.1). The eluted phages were mixed with 1 mL of E. coli ER2738 (A600 nm = 0.6) for 30 min at 37 °C, ampicillin was added to eliminate uninfected bacteria. The bacterial culture was infected again with 100 μL M13KO7 helper phage (~10^11^ CFU total) at 37 °C for 1 h and then added to 50 mL of 2X YT medium containing kanamycin 50 μg/mL and ampicillin 100 μg/mL overnight at 37 °C with vigorous shaking. The rescued phage library was precipitated with 20% polyethylene glycol/NaCl and resuspended in PBS. The concentrated phage solution was used for the next round of panning. After 2–3 rounds of a selection-amplification cycle, single colonies were randomly selected into a deep 96-well culture plate containing 850 μL/well of 2YT (100 μg/mL ampicillin) and shook at 37 °C for 3 h. 50 μL M13KO7 (~5 × 10^10^ CFU total) was then added to each well followed by the addition of 100 μL 2YT containing kanamycin (500 μg/mL) 1 h later. The clones were incubated at 37 °C with vigorous shaking overnight and then centrifuged at 3000 g for 10 min at 4 °C. 50 μL culture medium and 50 μL 5% PBST milk was added to each well of three 96-well Maxisorb immunoplates pre-coated with antigen (1 μg/mL) and blocked with 2% BSA in PBST. After 1 h incubation at room temperature, the plates were washed six times with PBST. 100 μL Protein M13-HRP antibody (1:3000) was added to each well for 1 h. the plates were washed six times with PBST buffer and twice with PBS, developed for 3 min with 3,3’,5,5’-tetramethyl-benzidine peroxidase substrate (Kirkegaard & Perry Laboratories), quenched with 1.0 M HCl and read spectrophotometrically at 450 nm. Positive clones were selected by the following criteria: ELISA OD450 > 0.2 for the antigen-coated well (antigen binding positive); OD450 < 0.1 for the negative control well. Unique clones were determined by sequencing the VHH DNA harbored in the phagemid.

### Screening of nanobodies blocking the interaction between RBD and hACE2

For blocking ELISA, a 96-well Maxisorp plate was coated with hACE2-Fc (2 μg/mL, 100 μL per well) at 4 °C, overnight and then blocked with blocking buffer (2% BSA in PBS) for 2 h. 50 ul of VHH-Fc cell supernatant (expression product of PCR fragment) was added to 50 uL of PBS, which contains RBD-his (40 ng/mL). VHH72-Fc and a non-related VHH-Fc (produced with the same method) were used as positive and negative controls, respectively. hACE2-Fc (2 ug/mL in PBS) was also used as a reference. After 1 h incubation with gentle shaking, 90 ul of the mixtures were transferred to the BSA-blocked plate and incubated for 20 min. RBD-His binding to the plate was detected with anti-His tag mouse monoclonal antibody (1:3000 dilution, 105327-MM02T, SinoBiological) and followed by an HRP conjugated anti-mouse IgG (H + L) Goat antibody (Cat. # A0216, Beyotime,). The RBD binding signals were developed by 3,3’,5,5’-tetramethyl-benzidine peroxidase substrate (Kirkegaard & Perry Laboratories), quenched with 1.0 M HCl and read at OD 450 nm by a spectrophotometer.

### RBD binding assays

For ELISA-based binding assay, the RBD-Fc antigens (0.2 µg per well) were coated in PBS buffer (pH7.4) on NUNC 96-well Maxisorb immunoplates overnight at 4 °C, then blocked with 5% skim milk in PBST for 1 h. 100 µL nanobodies prepared at serial concentrations in PBST with 2.5% milk were added to each well and incubated for 1 h under gentle shaking. The plate was washed with PBST and then added with 100 µL 1: 2000-diluted anti-human IgG conjugated with horse-radish peroxidase for another 1 h. The plates were washed with PBST buffer and twice with PBS, developed for 3 min with 3,3’,5,5’-tetramethyl-benzidine peroxidase substrate (Kirkegaard & Perry Laboratories), quenched with 1.0 M HCl and read spectrophotometrically at 450 nm. The EC_50_ was calculated according to Stewart and Watson method.

For HTRF-based binding assay, serially diluted nanobodies were mixed with 50 nM biotinylated RBD for 15 min at room temperature, then 6 μL mixtures were added to 4 μL 10 nM hACE2-Fc diluted in 384-well plate for additional 15 min. 10 μL of detection mixture containing Streptavidin-d2 (1:200, Cat. # 610SADLF, Cisbio,), and PAb Anti Human IgG-Eu cryptate (1:200, Cat. # 61HFCKLA, Cisbio) was finally added and incubated for 60 min. Signals were recorded by Envision (PerkinElmer) in FRET mode at wavelength of 665 nm. All reagents were diluted in assay buffer (PBS pH 7.4,10% BSA), and equilibrated at room temperature.

### Surface plasmon resonance (SPR) assay

The affinity of anti-RBD nanobodies and RBD antigen was measured with Biacore 8 K. A biosensor chip, Series S Sensor Chip Protein A (Cat. # 29127556, GE), was used to affinity capture a certain amount of Fc-tagged nanobodies to be tested and then flow through a series of COVID-19 S.P. RBD (Cat. # 40592-V08B, SinoBiological) under a concentration gradient on the surface of the chip (dilution ratio: 2, conc. levels: at least 5 (excluding curves with irregularities or high background)). Biacore 8 K (GE) was used to detect the reaction signal in real-time to obtain the binding and dissociation curves.

To measure the competitive response of anti-RBD nanobodies and hACE2, Fc-tagged nanobodies were immobilized on chip, then flowed with a 50 nM of RBD (Cat. # 40592-V08B, SinoBiological) and a 100 nM of hACE2 (Cat. #1010B-H08H, SinoBiological). the reaction signal in real-time were detected to obtain the binding and dissociation curves (theoretical ACE2 Rmax > 220 RU and kinetically simulated ACE2 binding > 160 RU for all). The buffer used in the experiment is an HBS-EP^+^ solution (pH 7.4, Cat. # BR100669, GE). The data obtained in the experiment was fitted with Biacore Insight Evaluation Software v3.0, GE software with a (1:1) binding model to obtain the affinity value.

### Pseudovirus neutralization

Pseudovirus neutralization assay was measured by reduction of Luciferase activity as described [48]. Briefly, pseudovirus bearing wildtype SARS-CoV-2 S protein or mutants were produced by co-transfection with plasmids expressing corresponding protein and backbone plasmid pNL-4-3-Luc.-R-E or VSV-dG-Luc. Pseudovirus was harvested, filtered and stored at −80 °C. The pseudoviral titer was measured as 50% tissue culture infectious dose (TCID50) by infection of BHK21 cells overexpressing human ACE2 according to the Reed-Muench method. Before infection of Caco-2 cells, pseudovirus was incubated with serial diluted nanobodies for 30 min at room temperature. Luciferase activity was measured after 72 h post-infection according to the manual of Bright-Glo™ Luciferase Assay System. Non-infected cells were considered as 100% inhibition, and cells only infected with the virus were set as 0% inhibition. The IC_50_ values were calculated with non-linear regression using Prism 5 (GraphPad Software, Inc., San Diego, CA, USA).

### SARS-CoV-2 neutralization assay

The SARS-CoV-2 (strain IVCAS 6.7512) was provided by the National Virus Resource, Wuhan Institute of Virology, Chinese Academy of Sciences. All SARS-CoV-2 live virus-related experiments were approved by the Biosafety Committee Level 3 (ABSL-3) of Wuhan University. All experiments involving SARS-CoV-2 were performed in the BSL-3 and ABSL-3 facilities. Briefly, nanobodies were serially diluted in culture medium and 100 μL was mixed with 100 μL (1000 PFU) SARS-CoV-2 for 30 min. The mixture was then added to Vero E6 cells in 48-well plates and incubated for 24 h, after which TRIzol (Invitrogen) was added to inactivate SARS-CoV-2 viruses and extract RNA according to the manufacturer’s instructions. First-strand cDNA was synthesized using the PrimeScript RT kit (TakaRa). A real-time quantitative PCR was used to detect the presence of SARS-CoV-2 viruses by the primers (Table 1). The relative number of SARS-CoV-2 viral genome copies was determined using a TaqMan RT-PCR Kit (Yeason). To accurately quantify the absolute number of SARS-CoV-2 genomes, a standard curve was prepared by measuring the SARS-CoV-2 N gene constructed in the pCMV-N plasmid. All SARS-CoV-2 genome copy numbers were normalized to GAPDH expression in the same cell.

**Table 1:** Primers.

SARS2-N-F	TAATCAGACAAGGAACTGATTA
SARS2-N-R	CGAAGGTGTGACTTCCATG
SARS2-N-P	GCAAATTGTGCAATTTGCGG
hGAPDH-F	CAGCCTCAAGATCATCAGCA
hGAPDH-R	TGTGGTCATGAGTCCTTCCA
hGAPDH-P	CTGCTTAGCACCCCTGGCCA
hACE2-F	CATTGGAGCAAGTGTTGGATCTT
hACE2-R	GAGCTAATGCATGCCATTCTCA
hACE2-P	CTTGCAGCTACACCAGTTCCCAGGCA
mGAPDH-F	TGCACCACCAACTGCTTAG
mGAPDH-R	GGATGCAGGGATGATGTTC
mGAPDH-P	CAGAAGACTGTGGATGGCCCCTC

### Protection of K18-hACE2 transgenic mice against SARS-CoV-2

K18-hACE2 transgenic mice expressing human ACE2 driven by the human epithelial cell cytokeratin-18 (K18) promoter [49], were purchased from Gempharmatech (heterozygous K18-hACE2 KI mice, #T037657, under C57BL/6J background) and housed in ABSL-3 pathogen-free facilities under 12-h light-dark cycles with *ad libitum* access to food and water. All animal experiments were approved by the Animal Care and Use Committee of Wuhan University. Age-matched (9 to10-week-old) female mice were grouped for infection of nanobodies (0.5 mg/kg). One day later, mice were inoculated with 6 × 10^4^ PFU of SARS-CoV-2 by the intranasal route. Body weights were monitored at 3 and 6 dpi. Animals were sacrificed at 3 or 6 dpi according to the protocol, and tissues were harvested for pathologic and histologic analysis. The investigator was blinded to the group allocation during the experiment.

### Plaque assay of lung tissue homogenates

The right lung was homogenized in 1 mL PBS using a Tissue Cell-destroyer 1000 (NZK LTD). Vero E6 (ATCC number: CRL-1586) cells were cultured to determine viral titer. Briefly, serial 10-fold serial dilutions of samples were added into monolayer cells. After adsorption at 37 °C, the virus inoculum was removed and cells were washed with PBS twice, then DMEM containing 5% FBS and 1% methylcellulose was supplemented. Plates were incubated for 2 days until obvious plaques can be observed. Cells were stained with 1% crystal violet for 4 h at room temperature. Plaques were counted and viral titers were defined as PFU/mL.

### Histological analysis

Lung samples were fixed with 4% paraformaldehyde, and paraffin embedded and cut into 3.5-mm sections. Fixed tissue samples were used for hematoxylin-eosin (H&E) staining and indirect immunofluorescence assays (IFA). Wuhan Pinuofei Biological Technology company provided the histological analysis service. For IFA, Anti-hACE2 antibody and anti-SARS-CoV/SARS-CoV-2 Nucleocapsid Antibody (Cat: 10108-RP01 and 40143-MM05, SinoBiological) were added as primary antibodies. The image information was collected using a Pannoramic MIDI system (3DHISTECH, Budapest) and FV1200 confocal microscopy (Olympus).

### Crystallization and structure determination of RBD-VHH60 complex

RBD (319–531) and VHH60 proteins were mixed at a molar ratio of 1:1.5 and further purified by Superdex 200 Increase 10/300 GL column with pH 7.5, 20 mM HEPES, 100 mM NaCl as running buffer after incubation on ice for 1 h. Crystal screening of the RBD-VHH60 complex at 8 mg/mL was performed by vapor-diffusion hanging-drop method at 18 °C. Diffracting crystals were obtained in the condition of 1.0 M Sodium acetate trihydrate and 0.1 M Sodium HEPES pH 7.5.

A diffraction data set with 3.40 Å was collected at BL19U1 beamline in the Shanghai Synchrotron Radiation Facility (SSRF) and auto-processed with autoPX software [50]. The RBD-VHH60 complex structure was determined by the molecular replacement method using Phenix [51] with previously reported SARS-CoV-2 RBD structure (PDB: 7VNB) [47] and VHH60 structure as previously described, and the atomic models were further refined and completed using Phenix 1.20 [51] and Coot 0.9 [52] respectively. Data collection, processing, and refinement statistics are summarized in Table S1. ChimeraX 1.6 [53] was used to generate the structural model figures.

### Cryo-EM sample preparation and data collection

The expression and purification procedure of SARS-CoV-2 WT S Protein was designed in our previous work [54]. Purified SARS-CoV-2 S protein was diluted to a concentration of 0.8 mg/mL in PBS, pH 7.4, and was incubated with nanobody VHH60 at a molar ratio of 1:3.6.

The mixture sample 3 μl was applied onto an H_2_/O_2_ glow-discharged, 300-mesh Quantifoil R1.2/1.3 gold grid (Quantifoil, Micro Tools GmbH, Germany). The grid was then blotted for 3 s with a blot force of 0 at 8 °C and 100% humidity and plunge-frozen in liquid ethane using a Vitrobot IV (Thermo Fisher Scientific, USA). Cryo-EM datasets were collected at a 300 kV Titan Krios microscope (Thermo Fisher Scientific, USA) equipped with a K3 detector (Gatan, USA). The exposure time was set to 2.4 s with a total accumulated dose of 60 electrons per Å [51], which yields a final pixel size of 0.84 Å. 5,155 micrographs were collected in a single session with a defocus range comprised between 1.2 and 2.2 μm using SerialEM. The statistics of cryo-EM data collection can be found in Table S2.

### Cryo-EM data processing

All dose-fractioned images were motion-corrected and dose-weighted by MotionCorr2 software [55] and their contrast transfer functions were estimated by cryoSPARC patch CTF estimation [56]. A total of 1,250,913 raw particles were auto-picked using the blob picker job and template picker job and the particles were extracted with a box size of 512 pixels, and the following 2D, 3D classifications, and refinements were all performed in cryoSPARC. 256,653 particles were selected after 2D classification, and these particles were used to do Ab-Initio reconstruction in five classes. Then these five classes were used as 3D volume templates for heterogeneous refinement with all selected particles, with 207,363 particles converged into a “1-up” conformation, the up RBD bound with VHH60 while the down RBDs remained in an apo form. Next, these particle sets were used to perform non-uniform refinement, yielding a resolution of 3.79 Å.

### Model building and refinement

To build the structures of the SARS-CoV-2 WT S-VHH60 structure, we used the Prefusion 2019-nCoV spike glycoprotein with a single receptor-binding domain up cryo-EM structure (PDB: 6VSB) [18] and the crystal structure of RBD-VHH60 complex as an initial model. This model was rigid body fitted into the cryo-EM map using the UCSF ChimeraX 1.6 [53] tool Fit in map. Then the model was manually built in Coot 0.9 [52] with the guidance of the cryo-EM electron density maps, and the overall real-space refinements were performed using Phenix 1.20 [51]. The data validation statistics are shown in Fig. S7 and Table S1. ChimeraX 1.6 [53] was used to generate the structural model figures.

### Data analysis

All the assays were conducted with at least duplicated biologic repeats. Results were presented by representative data or mean ± SEM with indicated numbers of replication. All data was analyzed by XLfit (IDBS, Boston, MA 02210) or Prism 5 (GraphPad Software, San Diego, CA 92108).

### Supplementary information

Supplementary information

## Data Availability

Data generated and analyzed in this study are provided in the Supplement Information. The coordinates and structure factor files for the SARS-CoV-2 WT S RBD-VHH60 complex and SARS-CoV-2 WT S -VHH60 complex have been deposited in the Protein Data Bank (PDB) under accession number 8XK2 (doi: 10.2210/pdb8xk2/pdb) and 8XKI (doi: 10.2210/pdb8xki/pdb), respectively.
