# Supplementary material for: A broad neutralizing nanobody against SARS-CoV-2 engineered from an approved drug
Source: Cell Death Dis. 2024 Jun 28;15(6):458. doi: 10.1038/s41419-024-06802-7 (PMC11211474; doi:10.1038/s41419-024-06802-7)
Supplement: Supplementary file 1 — Supplementary information [file 41419_2024_6802_MOESM1_ESM.docx]

**Supplementary information for**

**A broad neutralizing nanobody against SARS-CoV-2 engineered from the library based on an approved drug**

Qianyun Liu^1, #^, Yuchi Lu^2, 3, 4, #^, Chenguang Cai^5, #^, Yanyan Huang^5, #^, Li Zhou^6, 7^, Yanbin Guan^5^, Shiying Fu^5^, Youyou Lin^5^, Huan Yan^6^, Zhen Zhang^7^, Xiang Li^5^, Xiuna Yang^2, 4^, Haitao Yang^2, 4^, Hangtian Guo^8, *^, Ke Lan^6, 7, *^, Yu Chen^1, 6, *^, Shin-Chen Hou^5, *^, Yi Xiong^1, 5, 9, *^

Figure S1


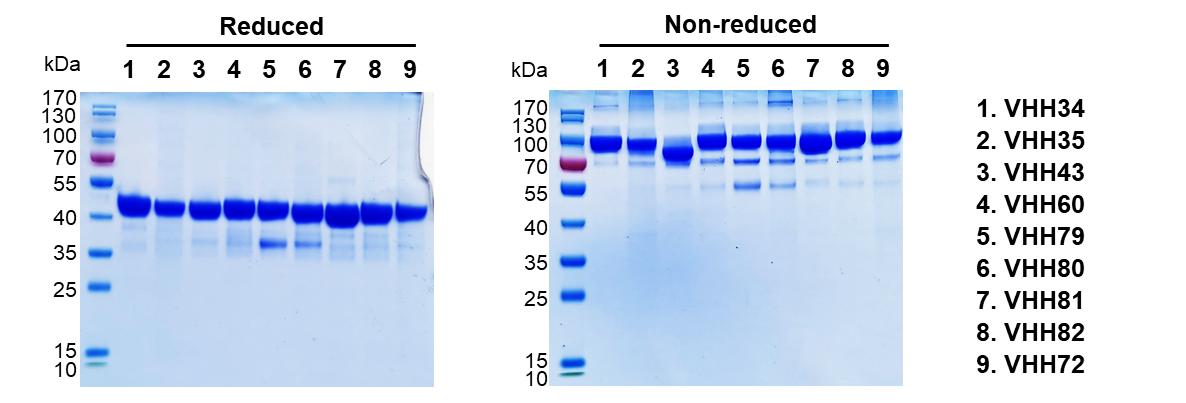


**Figure S1.** Purified nanobodies with Fc tag were subjected to SDS-PAGE. Left panel: protein sample was reduced by DTT; Right panel: non-reducing samples. Each lane was labeled with the number corresponding to the nanobodies at far right.

Figure S2


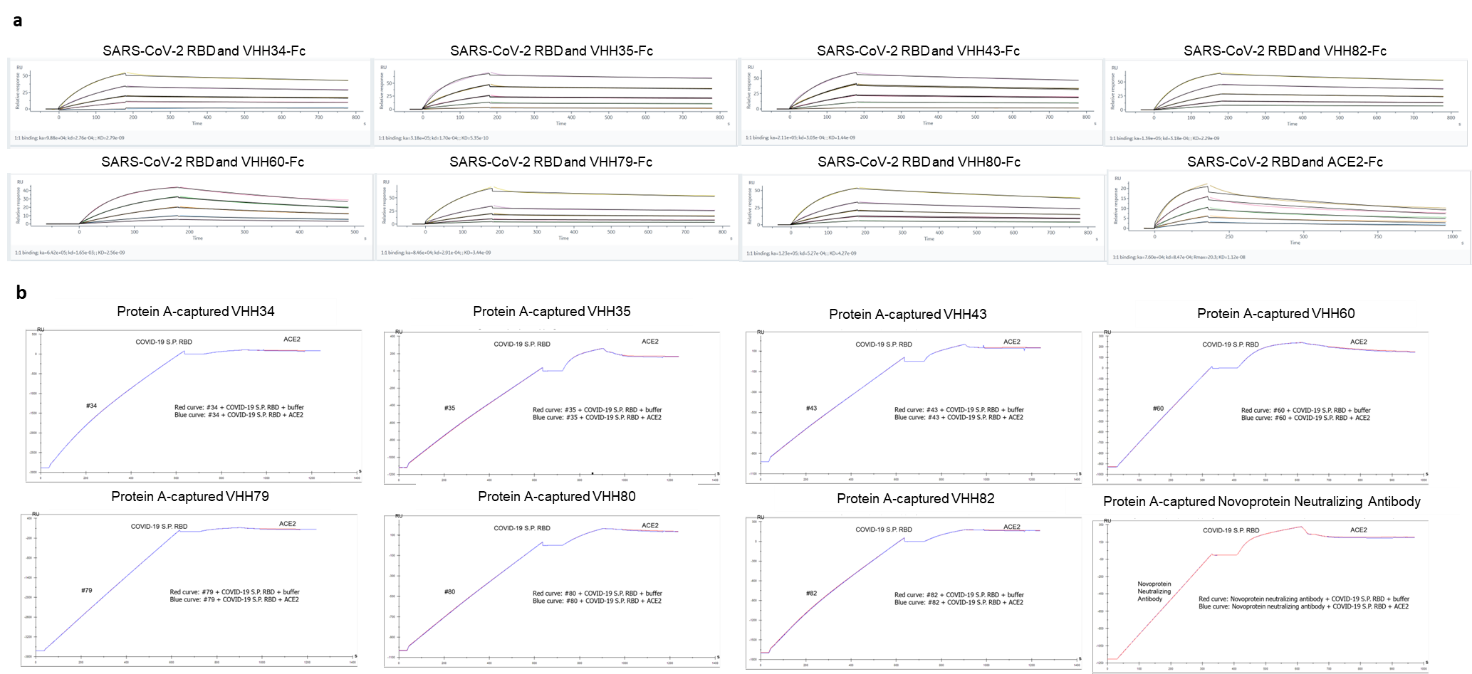


**Figure S2.** **a.** Affinity between RBD and Nanobodies were measured by SPR. Fc tagged nanobodies and ACE-2 were captured onto the Protein A Chip, then RBD flew through with highest concentration for each protein as indicated: VHH34 (100 nM), VHH35 (50 nM), VHH43 (50 nM), VHH60 (25 nM), VHH79 (100 nM), VHH80 (100 nM), VHH82 (100 nM), hACE2-Fc (200 nM). **b**, Nanobodies inhibit interaction between RBD and hACE2 by SPR. The Fc tagged nanobodies and a reference antibody (Novoprotein Neutralizing Antibody) were captured onto the Protein A Chip as indicated at the first curve. The second binding curve was detected when 50 nM RBD (S.P.RBD) was injected. Lastly, injection of 100 nM hACE2 showed no further binding curve in all the experiments.

Figure S3

**Figure S3.** VHH35 inhibits pseudovirus carrying wildtype spike protein infection on Caco-2 cell line. The value of IC_50_ is the average of two independent experiments.

Figure S4

**Figure S4.** Scheme of animal challenge. Total 10 mice were separated in each group, 5 mice were sacrificed 3 d.p.i. all remaining mice will be terminated after meeting certain criteria.

Figure S5

**Figure S5.** Trimeric VHH60 Inhibits interaction of RBD and hACE2 by HTRF.

Figure S6

**Figure S6.** Trimeric VHH60 inhibits pseudovirus carrying wildtype spike protein infection on Caco-2 cell line. The value of IC_50_ is the average of two independent experiments.

Figure S7


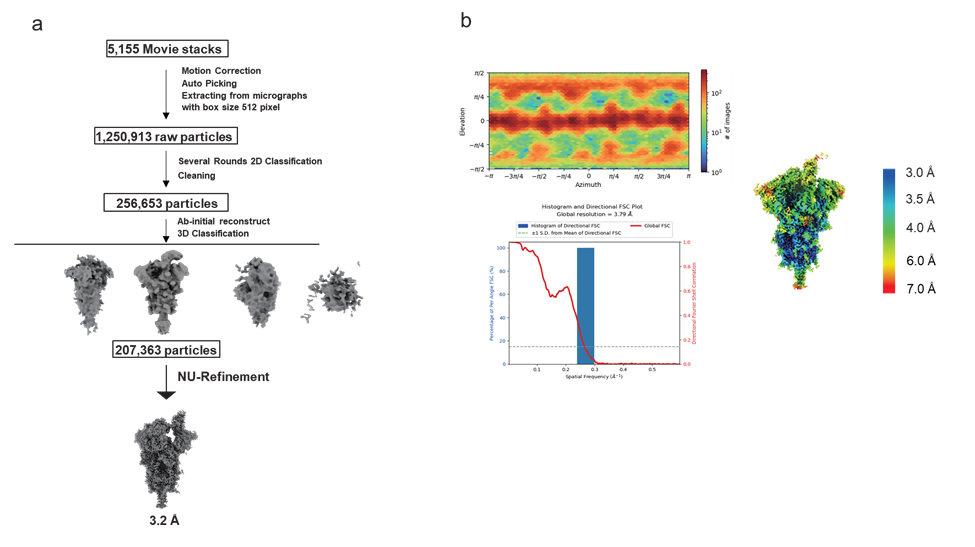


**Figure S7.** Cryo-EM data processing for the Omicron BA.2 Spike protein and human ACE2 complex. **a**, Workflow for the Omicron BA.2 Spike-BAT2022 3D Reconstructions. **b**, The viewing direction distribution plot, gold-standard FSC curves with the 0.143 cutoff indicated by a horizontal blue line, Global FSC and Histogram, and cryo-EM maps colored by local resolution.

Figure S8


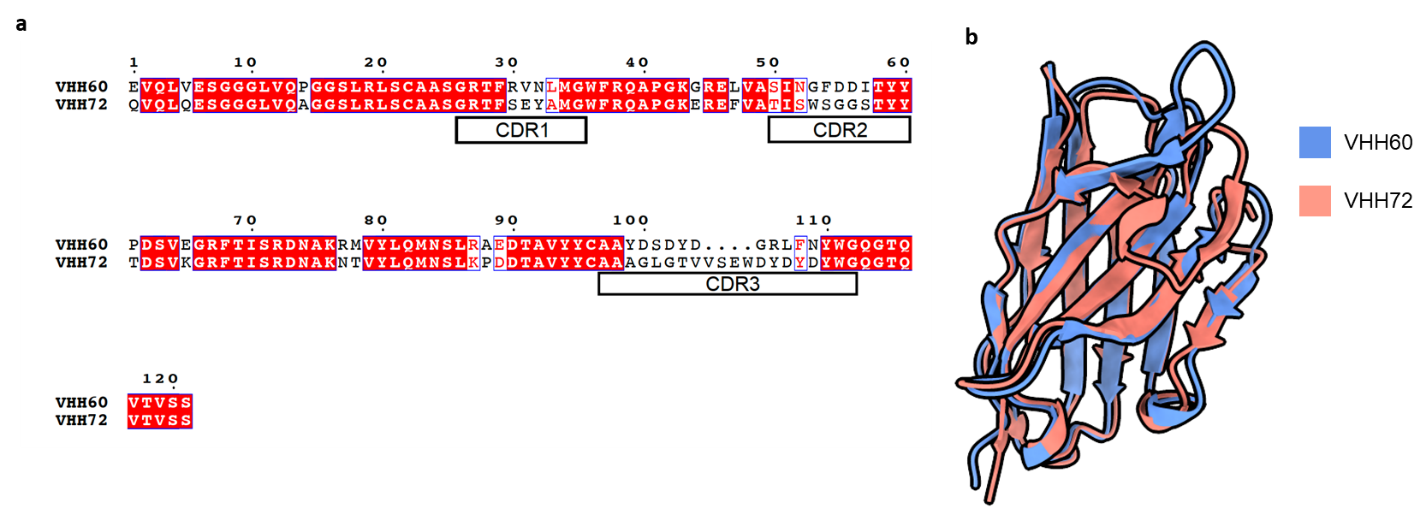


**Figure S8**. **a**, Sequence alignment of VHH60 and VHH72, aligned by Clustal Omega, the figure is made by ESPript 3.0 (https://doi.org/10.1093/nar/gku316). **b**, Structure alignment sequence alignment of VHH60 (cornflower) and VHH72 (salmon), aligned by Matchmaker in ChimeraX 1.6 (doi:10.1002/pro.3943).

**Table S1**, Cryo-EM data collection, models refinement and validation statistics of the Spike-VHH60 datasets.

| **Data collection and processing** | VHH60 + spike  “1-up” conformation |
| --- | --- |
| **Voltage (kV)** | 300 |
| **Detector** | K3 |
| **Pixel size (Å)** | 0.84 |
| **Electron dose (e^-^/ Å^2^)** | 60 |
| **Defocus range** | -1.2 to -2.2 |
| **Final particles** | 207,363 |
| **Final resolution (Å)** | 3.79 |
| **Model refinement** |  |
| **Map-model CC (mask)** | 0.76 |
| **Initial model used** | 6VSB |
| **RMSD** |  |
| Bond lengths (Å) | 0.003 |
| Bond angles (°) | 0.551 |
| Molprobity score | 1.74 |
| Clash score | 6.22 |
| Rotamer outliers (%) | 0.23 |
| Cβ outliers (%) | 0.00 |
| CaBLAM outliers (%) | 3.79 |
| **Ramachandran statistics** |  |
| Favored (%) | 94.06 |
| Allowed (%) | 5.84 |
| Outliers (%) | 0.10 |

**Table S2**, Crystal data collection and refinement statistics.

| **Wavelength** | 0.9785 |
| --- | --- |
| **Resolution range** | 31.21- 3.4 (3.49 - 3.4) |
| **Space group** | P 2 2 21 |
| **Unit cell** | 80.85 100.61 227.43 90 90 90 |
| **Total reflections** | 52492 (3956) |
| **Unique reflections** | 23631 (1567) |
| **Multiplicity** | 2.0 (2.0) |
| **Completeness (%)** | 90.01 (79.22) |
| **Mean I/sigma(I)** | 5.96 (2.00) |
| **Wilson B-factor** | 52.90 |
| **R-merge** | 0.09947 (0.377) |
| **R-meas** | 0.1407 (0.5331) |
| **R-pim** | 0.09947 (0.377) |
| **CC1/2** | 0.988 (0.815) |
| **CC*** | 0.997 (0.948) |
| **Reflections used in refinement** | 23631 (1567) |
| **Reflections used for R-free** | 1803 (119) |
| **R-work** | 0.2560 (0.3442) |
| **R-free** | 0.3012 (0.4103) |
| **CC (work)** | 0.906 (0.743) |
| **CC (free)** | 0.891 (0.678) |
| **Number of non-hydrogen atoms** | 9414 |
| macromolecules | 9414 |
| ligands | 0 |
| solvent | 0 |
| **Protein residues** | 1201 |
| **RMS (bonds)** | 0.002 |
| **RMS (angles)** | 0.51 |
| **Ramachandran favored (%)** | 93.70 |
| **Ramachandran allowed (%)** | 5.87 |
| **Ramachandran outliers (%)** | 0.43 |
| **Rotamer outliers (%)** | 0.60 |
| **Clashscore** | 1.75 |
| **Average B-factor** | 67.53 |
| **macromolecules** | 67.53 |
